# Migration of repetitive DNAs during evolution of the permanent translocation heterozygosity in the oyster plant (*Tradescantia* section *Rhoeo*)

**DOI:** 10.1007/s00412-022-00776-1

**Published:** 2022-07-27

**Authors:** Hieronim Golczyk, Eva Hřibová, Jaroslav Doležel, Ángeles Cuadrado, Frauke Garbsch, Stephan Greiner, Monika Janeczko, Marek Szklarczyk, Maciej Masłyk, Konrad Kubiński

**Affiliations:** 1 Department of Molecular Biology, Institute of Biological Sciences, John Paul II Catholic University of Lublin, Konstantynów 1i, 20-708 Lublin, Poland; 2 Institute of Experimental Botany of the Czech Academy of Sciences, Centre of the Region Hanaá for Biotechnological and Agricultural Research, Šlechtitelů 31, 77900 Olomouc, Czech Republic; 3 Department of Biomedicine and Biotechnology, University of Alcalá, 28871 Alcalá de Henares, Madrid Spain; 4 Max Planck Institute of Molecular Plant Physiology, Am Mühlenberg 1, 14476 Potsdam-Golm, Germany; 5 Department of Plant Biology and Biotechnology, University of Agriculture in Krakow, Al. 29 Listopada 54, 31-425 Cracow, Poland

**Keywords:** Inversions, Permanent translocation heterozygosity, Rabl configuration, Repetitive DNA, *Tradescantia spathacea*, Translocations

## Abstract

Due to translocation heterozygosity for all chromosomes in the cell complement, the oyster plant (*Tradescantia spathacea*) forms a complete meiotic ring. It also shows Rabl-arrangement at interphase, featured by polar centromere clustering. We demonstrate that the pericentromeric regions of the oyster plant are homogenized in concert by three subtelomeric sequences: 45S rDNA, (TTTAGGG)n motif, and TSrepI repeat. The Rabl-based clustering of pericentromeric regions may have been an excellent device to combine the subtelomere-pericentromere sequence migration (via inversions) with the pericentromere-pericentromere DNA movement (via whole arm translocations) that altogether led to the concerted homogenization of all the pericentromeric domains by the subtelomeric sequences. We also show that the repetitive sequence landscape of interstitial chromosome regions contains many loci consisting of *Arabidopsis*-type telomeric sequence or of TSrepI repeat, and it is extensively heterozygous. However, the sequence arrangement on some chromosomal arms suggest segmental inversions that are fully or partially homozygous, a fact that could be explained if the inversions started to create linkages already in a bivalent-forming ancestor. Remarkably, the subterminal TSrepI loci reside exclusively on the longer arms that could be due to sharing sequences between similarly-sized chromosomal arms in the interphase nucleus. Altogether, our study spotlights the supergene system of the oyster plant as an excellent model to link complex chromosome rearrangements, evolution of repetitive sequences, and nuclear architecture.

**Supplementary Information:**

The online version contains supplementary material available at 10.1007/s00412-022-00776-1.

## Introduction

**Fig. 1:**
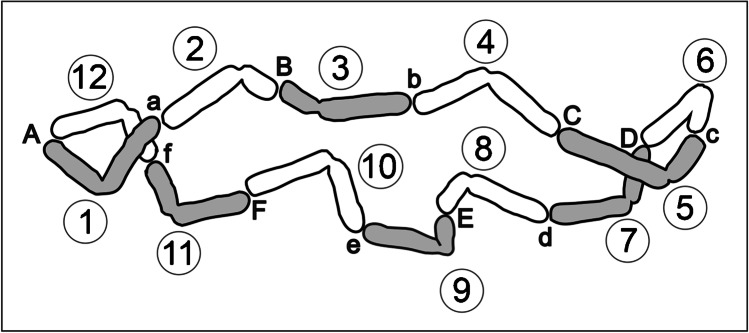
Meiotic ring of the oyster plant. The conjoining chromosomal ends/arms are labeled with the same letter, the chromosomes are numbered from 1 to 12 (starting from chromosome Aa), and the α or β complex is gray or white, respectively. Note that chromosomes 2–3, 5–6, 8–9, and 11–12 conjoin by their shorter arms. Chromosome morphology according to Golczyk et al. (2010)

Oyster plant (*Tradescantia spathacea* Sw. Stearn) from the monotypic *Rhoeo* section of the *Tradescantia* genus is characterized by permanent translocation heterozygosity (PTH) (Belling 1927; Sax 1931), a fascinating genetic system found also in some other plants (Holsinger and Ellstrand 1984) including *Oenothera* species (Cleland 1972; Golczyk et al. 2014). This system has probably evolved as the ultimate way to maintain heterozygosity in face of inbreeding depression in those rare plant taxa who had been able to incorporate extensive reciprocal translocations as a stable part of their genetic system (Holsinger and Ellstrand 1984). Permanent translocation heterozygosity is featured by meiotic rings at metaphase I where each chromosome is attached to its two neighbors by terminal chiasmata (Cleland 1972; Loidl 2016; Wielstra 2020; Holsinger and Ellstrand 1984; Golczyk et al. 2008; Berdan et al. 2021). PTH rings result from reciprocal translocations and are permanent due to elimination of homozygous progeny (Cleland 1972; Holsinger and Ellstrand 1984). In the ring that is formed at the first meiotic division, every chromosome is partially homologous to its two neighbors and kinetochores are alternately orientated (Cleland 1972; Holsinger and Ellstrand 1984). Thus, every second chromosome goes to the same pole. In this way, co-segregating alternate chromosomes form a paternal or maternal group, a so-called Renner complex, designated α or β, acting as a basic hereditary unit (Rauwolf et al. 2008; Hollister et al. 2019). Since homologous recombination of the PTH complexes is virtually undetectable and during anaphase I, both complexes segregate as whole entities, each of them is viewed as a superlinkage (Brown and Levin 2011; Rauwolf et al. 2008; Hollister et al. 2019). For a better explanation, the chromosomal arrangement in the meiotic ring of the oyster plant is graphically depicted and explained in Fig. 1.

So far, molecular cytogenetics has provided only limited insights into the structure of the PTH karyotypes and their evolution in plants. Briefly, the main achievement is the FISH (fluorescence in situ hybridization) mapping of the 5S and 18S-5,5S-26S ribosomal RNA gene arrays (rDNA) in oyster plants (Golczyk 2011b; Golczyk et al. 2005, 2010) and in *Oenothera* species (Golczyk et al. 2014). Unlike *Oenothera*, each of the chromosomal arms of the meiotic ring in the oyster plant can be identified at mitotic metaphases using double-target FISH with rDNA (5S and 45S) probes combined with detailed morphometric analysis (Golczyk et al. 2005, 2010). Thus, the structure of chromosome arms that conjoin in the meiotic ring can be studied using cytologically much preferable mitotic material.

A hypothesis for the PTH evolution in the oyster plant is that chromosomal rearrangements have occurred that preserve the length similarity between the arms that conjoin in the meiotic ring (reviewed in: Golczyk 2013). These arms are in general well matched in length (Fig. 1), which is, however, not required given that the chromosomal ends are the exclusive sites of homologous recombination and effective pairing in the PTH ring (Rauwolf et al. 2008). Consequently, ectopic exchanges between pericentromeric regions resulting in whole-arm translocations have been suggested as a mechanism leading to meiotic catenation (Golczyk 2013; Golczyk et al. 2010). Such exchanges could have been facilitated by the so-called Rabl configuration (Rabl 1885; Pouokam et al. 2019), a remnant of the anaphase orientation of chromosomes from the preceding mitoses that is featured by polar clustering/association of the centromeres at interphase (Therizols et al. 2010). Indeed, it was well-documented that the polarized centromere association occurs on a large scale in cycling meiotic and somatic cells and during development in oyster plant (Golczyk et al. 2020). However, whole-arm inversions due to breakpoints at subtelomeric and pericentromeric area seem another interesting evolutionary scenario for verification (Golczyk 2011b, 2013; Golczyk et al. 2010). If both whole-arm translocations and whole-arm inversions occurred, subtelomere-pericentromere migration (via whole-arm inversions) of subtelomeric repetitive DNA sequences and their concerted spread across pericentromeric regions (due to pericentromere clustering and whole-arms translocations) should be expected. The possibility for such a complex genome-wide pattern of sequence migration cannot be excluded since low-stringency FISH with 45S rDNA probe generated signals in the pericentromeric regions at mitosis (Golczyk et al. 2010). However, so far, standard high stringency FISH on mitotic chromosomes of PTH clones has allowed visualization of exclusively subtelomeric 45S rDNA (Golczyk et al. 2005, 2010). Thus, to test the above hypothesis on the genome-wide sequence migration, the question whether pericentromeres of mitotic chromosomes have been homogenized by subtelomeric 45S rDNA should be unambiguous answered. However, equally important is also to check if other subtelomeric sequences including (TTTAGGG)n motif are present in the pericentromeric regions.

Here, our goal was to test the hypothesis on the above genome-wide repetitive sequence migration by studying the repetitive sequence landscape on mitotic chromosomes. Our results support this hypothesis by detecting the pericentromeric 45S rDNA FISH signals on mitotic chromosomes at standard stringency and by showing that two other types of repetitive DNA sequences, i.e., (TTTAGGG)n telomeric motif and a newly isolated semitandem repeat, are also shared between pericentromeric and subtelomeric domains, and that all the three subtelomeric sequences had homogenized pericentromeric regions. Interestingly, our chromosomal map showing the highest density of cytogenetic markers for any PTH system demonstrates for the first time a high level of structural heterozygosity of the chromosomal arms that conjoin in the meiotic ring. Our map suggests also the occurrence of intercalary inversions and raises the possibility of reducing recombination by inversions already in a bivalent-forming ancestor, as a preparatory step towards PTH. Interestingly, we show that the subterminal TSrepI loci reside exclusively on the longer arms that could be due to migration of sequences by physical contact between similarly sized chromosomal arms within the Rabl-interphase arrangement. Altogether, our study spotlights the supergene system of the oyster plant as an excellent plant model for multidimensional research to link complex chromosome rearrangements, evolution/function of repetitive sequences, and nuclear architecture.

## Materials and methods

### Plant material

The described arrangement of all the FISH foci (“Results”) was studied for 14–17 plants belonging to each of the three previously studied ring-forming clones (Golczyk et al. 2005). Additionally, five plants from each clone were used to generate averaged chromosomal measurements. All the plants were grown in pots filled with soil in a greenhouse at 25–27 °C. To induce rooting, the stem cuttings were kept in glass jars filled with fresh tap water and wrapped with aluminum foil. Vigorously growing ca. 2–3-cm-long adventitious roots were excised, pre-treated with a saturated solution of α-bromonaphtalene, fixed in 3:1 ethanol-glacial acetic acid, and stored at − 20 °C until required.

### Molecular probes and fluorescence in situ hybridization (FISH)

A clone containing the 427 bp semitandemly organized TSrepI.01 sequence, referred here to as TSrepI (Data Set S1; Fig. S1), was isolated and analyzed as described for banana by Hřibová et al., (2010). Briefly, genomic DNA of *T.spathacea* was mechanically sheared into 500–1000-bp fragments that were ligated into pCR-XL-TOPO vector containing M13-type flanking sequences and transformed into One Shot TOP10 electrocompetent *Escherichia coli* (Thermo Fischer Scientific/Invitrogen Life Technologies, Carlsbad, USA). The clones were plated onto bioassays and recombinant colonies were picked by GeneTACTM G3 workstation (Genomic Solutions) into 96-well plates. Then the clones were screened for the presence of highly repetitive sequences by spotting onto Hybond-N + filters (Amersham) and by probing with the total genomic DNA of *Tradescantia spathacea*, as well as with 45S and 5S rDNA. The TSrepI.01 clone always produced a remarkably dark and strong hybridization signal with the total genomic DNA and the lack of thereof when probed with rDNAs, which was interpreted as a putative highly repeated DNA sequence other than rDNA. The clone was sequenced using the BigDye Terminator v3.1 Cycle Sequencing kit (Applied Biosystems, Foster City, USA) and run on ABI 3730xl DNA analyzer (Applied Biosystems, Foster City, USA). The nucleotide sequence was edited using Staden Package (Staden 1996) and checked for tandem organization by dotter analysis (Sonnhammer and Durbin 1995).

The TSrepI sequence was PCR-labeled with M13 primers (Genomed, Poland) in the presence of biotin-16-dUTP (Biotin-dUTP, Roche) or digoxigenin-11-dUTP (DIG-dUTP, Roche). The 5S rDNA-specific probe was generated by PCR with tetramethyl-rhodamine-5-dUTP (TRITC-dUTP, Roche) as reported previously (Golczyk 2019; Golczyk et al. 2010). The 2,3 kb *Cla*I fragment of the 25S rDNA of *Arabidopsis thaliana* (Unfried and Gruendler 1990) labeled via nick-translation with DIG-dUTP (Roche) or TRITC-dUTP, or biotin-dUTP (Roche) was used for the detection of 18S-5,8S-25S ribosomal gene arrays (45S rDNA). The deoxyribonucleotide (AC)_10_ oligomer was synthesized with Dy547 at either end (Isogen Life Science, De Meern, The Netherlands). Three sensitive *Arabidopsis*-type telomeric probes were tested: (i) double-stranded DNA concatameres generated by the non-template PCR (Ijdo et al. 1991) and converted into ca. 100 bp dsDNA fragments by nick translation in the presence of DIG-dUTP (Roche); (ii) synthetic 63-mer (5’-CCCTAAA-3’)_9_, or (iii) 21-mer (5’-CCCTAAA-3’)_3_ single-stranded DNA labeled at both extremes with Dy547 or DIG-dUTP (Isogen Life Science, De Meern, The Netherlands). The 21-mer telomeric probe and (AC)_10_ oligonucleotide were applied exclusively for the non-denaturating FISH (ND-FISH) procedure carried out according to Cuadrado et al. (2009). The material was stored in the fixative only for 1 day and only freshly made preparations were used for hybridization.

The remaining probes were used for the standard FISH or EC-FISH (ethylene-carbonate FISH) in dual-target combinations as already described (Golczyk 2019; Golczyk et al. 2010). The digoxigenin and biotin were detected using antidigoxigenin FITC-conjugated antibody (Roche) and streptavidin-Cy3/-Texas Red (Sigma) or streptavidin Alexa Fluor 488 conjugates (Fisher Scientific), respectively. The improvement of the rDNA signal specificity and sensitiveness at standard stringency was achieved when the following requirements were met: (a) storing the material in the fixative for only one-two days, (b) additional maceration of the preparations in 60% acetic acid on a hot plate at 75 °C as described previously (Golczyk 2019); (c) using only freshly-made preparations; (d) applying biotin-streptavidin detection.

### Microscopy techniques and data analysis

Microscope observations were carried out with the Nikon Eclipse Ni-U epifluorescence microscope under 100 × and 60 × planachromatic immersion objectives. Extended-depth-of-focus (EDF) images were obtained by capturing 10–15 different focal planes of the same object by a cooled monochrome DSQi1 camera (Nikon), stacking them and combining using EDF function of the NIS Elements software (Laboratory Imaging, Ltd.). The intrachromosomal (TTTAGGG)_n_ signals can be generated by all FISH-techniques and all telomeric probes, but they quickly fade, which requires finishing acquisition within 1–2 days after mounting the preparations. The superimposing and uniform image processing of the FISH-images and chromosome measurements were performed using Adobe Photoshop (Adobe Systems) and ImageJ software (Fiji package, https://imagej.net/Fiji/Downloads). Six best mitotic metaphases from each plant were added to the pool for statistical calculations (in total 90 metaphase plates), which were done with Microsoft Excel 2016.

## Results

### Heterochromatic pericentromeres and selectively shared DNA repeats

**Fig. 2:**
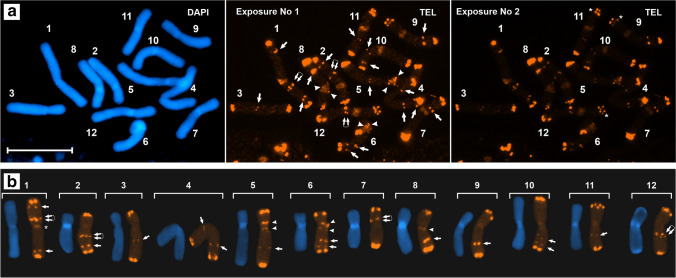
FISH**-**mapping of intrachromosomal telomeric sites; 1–12, individual chromosomes. DAPI staining in blue color. Telomeric sites in orange. Bars = 10 µm. Dispersed pericentromeric signals on all chromosomes except chromosome 1 (asterisk) tended to fade in **b**. Arrowheads, interstitial (TTTAGGG)_n_ loci; double arrows, (TTTAGGG)n loci duplications; arrowheads, (TTTAGGG)_n_ cluster concentrations (bands) on the heterochromatin periphery. **a** ND-FISH with the synthetic (TTTAGGG)_3_ probe. The asterisks in the right panel indicate 2–3 (TTTAGGG)_n_ distinct loci within distal chromosome regions that could be resolved in this metaphase plate. **b** Standard FISH with the mixture of ca. 100 bp dsDNA fragments of the (TTTAGGG)_n_ array used as a probe on individual chromosomes

**Fig. 3:**
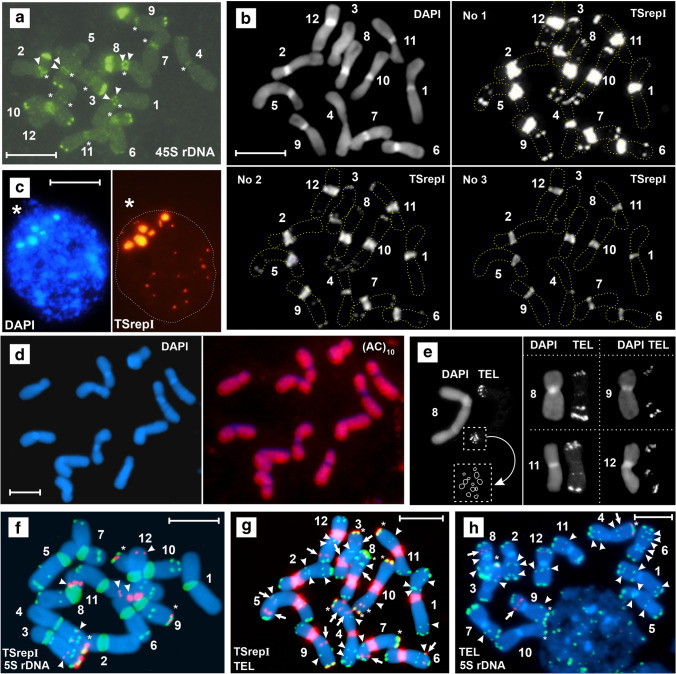
FISH**-**mapping of the repetitive DNAs. **a** All pericentromeric regions display the presence of 45S rDNA at standard stringency. Arrowheads, 45S rDNA clusters concentrated on the heterochromatin periphery as bands. Asterisks, centromeric regions. **b** TSrepI sequence arrangement under long (no. 1), moderate (no. 2), and short (no. 3) camera exposure. The FISH-exposure patterns indicate that the pericentromeric regions are heavily but rather uniformly loaded with the TSrepI sequence, but much more abundantly than in the subtelomeric and interstitial chromosomal sites. **c** Pericentromeric regions positive for DAPI (left panel) and for strong TSrepI-FISH signals (right panel) typically cluster into collective chromocenters at one pole at interphase. The remaining TSrepI sites are located some distance apart towards the opposite nuclear hemisphere (right panel). The asterisk marks the centromere pole. **d** The (AC)_10_ oligonucleotide (red) is generally absent or very rare in pericentromeric heterochromatin. **e** EC-FISH with the mixture of ca. 100 bp dsDNA fragments of the (TTTAGGG)_n_ array. Chromosome arms may possess from two big (arm 11F – right panel) to twelve smaller (arm 8d – left panel, with graphical interpretation) punctate (TTAGGG)_n_ foci at their subtermini, depending on the degree of contraction (compare pictures of chromosome 8 in the two panels). **(f)** Standard FISH with TSrepI (green) and 5SrDNA (red) probes. The arrowheads mark interstitial 5S rDNA sites, and the asterisks point to subterminal sites where both repeats are adjacent. The duplicated 5S rDNA loci on chromosomal arms 8E-9E tend to fuse, forming bigger joint clusters. **g** Standard FISH with TSrepI (red) and the 100 bp dsDNA telomeric arrays (green) as probes. The arrowheads or arrows point to interstitial (TTTAGGG)_n_ or TSrepI sites, respectively. The asterisks mark subtelomeric sites where the two repeats are adjacent. **h** EC-FISH with 5S rDNA (red) and 63-mer telomeric oligonucleotide (green) as probes. The arrowheads or arrows point to interstitial (TTAGGG)_n_ or 5S rDNA or sites, respectively. The asterisks mark subtelomeric sites where the two repeats are adjacent

**Fig. 4:**
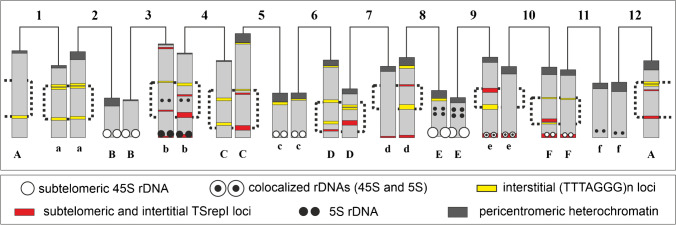
Repetitive DNA sites on chromosomes at meiosis. The intercalary sections are dot-outlined. They could be distinguished when at least two well distanced interstitial loci could be detected on one or on both conjoining arms. The distances between intercalary sections and subtelomeric regions mark distal sections

Fluorescence in situ hybridization (FISH) revealed the presence of the telomeric (TTTAGGG)_n_ motif and 45S rDNA in (su)btelomeric and pericentromeric regions. In addition, a multiplicity of telomeric loci along the chromosomal arms and their positional variability were uncovered. The chromosomal position of FISH signals is shown in Figs. 2, 3a and b, 3e and h, and 4. Pericentromeric telomere sequence signals and signals of 45S rDNA could be seen evenly dispersed across the heterochromatin and as distinct peripheral bands (Figs. 2 and 3a). The latter clearly correspond to the previously revealed pericentromeric chromomycin A_3_ bands (Golczyk et al. 2010). Whether they reflect a trend for a regional modification of the amount of repetitive DNAs around breakpoint sites (Eichler 1999; Bulazel et al. 2007), remains to be explored.

The third type of repeated DNA that is shared by subtelomeres and pericentromeric heterochromatin is short-degenerated semi-tandemly arranged motifs, as shown for the newly cloned sequence designated TSrepI, which contains these motifs (“Methods,” Fig. 3b and c, Data Set S1 and Fig. S1). However, pericentromeres also differ from the subtelomeric domains and from the rest of chromosome regions in that they lack detectable 5S rDNA (Fig. 3f) and CA/GT dinucleotide repeat sequence clusters (Fig. 3d). This may reflect an independent molecular evolution of the subtelomeric and pericentromeric region via sequence elimination/degeneration, counterbalancing the subtelomere-pericentromere homogenization.

### The repetitive sequence landscape along chromosomal arms can now be revealed

Mapping of the numerous (TTTAGGG)_n_, TSrepI, and rDNA loci (Fig. 3f–h as examples) has shown that repetitive sequences tend to occupy different niches along the chromosomal arms (Fig. 4). Notably, making copies of clusters manifests as interstitial duplicated (TTAGGG)_n_ loci on arms 1a, 2a, 7D, and 12A (Figs. 2 and 4), as well as the 5S rDNA locus duplication on chromosome arms 8E and 9E (Figs. 3f and 4, also see Golczyk et al. 2010). Eight pairs of intercalary chromosomal sections were distinguished (Fig. 4) whose size is 22% to 61% of the arm length (Table S1), thus representing a substantial fraction of euchromatin. A high degree of structural heterozygosity was revealed, since within each of such five pairs (localized on arms 4C–5C, 7d–8d, 9e–10e, 10F–11F, and 12A–1A), sections differ in their structure (Fig. 4). However, some of the conjoining arms express structural homology or its remnants. Arms 1a and 2a appear to have their Sects. (43–51% of arm length) perfectly homologous in terms of the (TTTAGGG)_n_ sequence arrangement and arms 3b–4b (39–43%) show incomplete structural homology of their sections. The most striking is that the distance between the distal and interstitial 5S rDNA locus (ca. 40% of arm length) in both “b” arms is the same. Another feature of the PTH karyotype indicative of structural homologies is that, within each of the five arm pairs 1a–2a, 3b–4b, 4C–5C, 6D–7D, and 10F–11F, the most proximal (TTTAGGG)_n_ loci are equally or similarly distanced from the chromosome termini (Fig. 4).

### TSrepI repeat invasion into non-pericentromeric sites is related with the size of chromosomal arms

As previously shown (Golczyk et al. 2010), fifteen long (1A, 1a, 2a, 3b, 4b, 4C, 5C, 6D, 7d, 8d, 9e, 10e, 10F, 11F, 12A) and nine short (2B, 3B, 5c, 6c, 7D, 8E, 9E, 11f, 12f) chromosome arms can be distinguished in the PTH karyotype of the oyster plant (Fig. 4). It is then striking that all the eight TSrepI distal loci occupy exclusively long arms (arms: 3b, 4b, 7d, 8d, 9e, 10e, 10F, 11F). Even on chromosome 4, whose long arms are somewhat unequal in length (Golczyk et al. 2010), the TSrepI distal locus resides on the longer arm (Fig. 4). On the other hand, the long arms of chromosome 10 are strictly equal in length (Golczyk et al. 2010) and each is equipped with the TSrepI distal locus (Fig. 4). Thus, if a chromosome has arms even slightly different in length, the distal TSrepI locus (if present) is located on the longer arm. It has already been statistically established by Golczyk et al. (2010) that mismatches in length between conjoining chromosome arms in the ring exist for six arm positions, the rest of the conjoining arms being equal in length. These length differences can be expressed in a gradient, starting from the highest value: 1.79% of karyotype length for 6D–7D > 1.67% for 4C–5C > 0.86% for 1a–2a > 0.61% for 12A–1A > 0.56% for 9e–10e > 0.55% for 7d–8d (Golczyk et al. 2010). Thus, the lack of distal TSrepI loci on the remaining long arms (Fig. 4) is inherent for those arm pairs whose members do not exactly fit in length (1a–2a, 12A–1A) or are vastly uneven in length (6D–7D, 4C–5C).

### Subtelomeres that conjoin in the meiotic ring are complex and homologous at the sequence level

Distal chromosome regions of metaphase chromosomes display 2–12 punctate (TTTAGGG)_n_ fluorescence foci, which means that approximately up to six distinct telomeric loci can be present (Figs. 2a right 3e). Such duplications of (TTAGGG)_n_ subtelomeric loci were also found in the closely related *Tradescantia virginiana*, which also forms terminal chasmata at metaphase I of meiosis (Golczyk 2011a). Notably, if a chromosomal arm has (sub)terminally located rDNA (5S and/or 45S) and/or TSrepI clusters, the adjacent arm of the neighboring chromosome in the meiotic ring has always the same composition of the relevant region (arm pairs: 2B–3B, 3b–4b, 5c–6c, 7d–8d, 8E–9E, 9e–10e, 10F–11F, 11f–12f (Fig. 4)). Furthermore, there are five unique sequence combinations specific for a given pair of arms: 3b–4b (TSrepI + 5S rDNA + telomeric motif), 7d–8d (TSrepI + telomeric motif), 9e–10e (TSrepI + 5S rDNA + 45S rDNA + telomeric motif), 10F–11F (TSrepI + 45S rDNA + telomeric motif), 11f–12f (5S rDNA + telomeric motif).

## Discussion

### Identification of chromosomal arms and studying their structure at mitosis appears now to be easy

So far, chromosome arm identification in *Tradescantia spathacea* at mitosis has been possible but troublesome and required much experience since being possible only when the double-target FISH with the two rDNA probes (5S rDNA and 45S rDNA) was combined with morphometric analysis (Golczyk et al. 2005). We showed here for the first time that each of the chromosomal arms of the meiotic ring in the oyster plant can be unambiguously and quickly identified at mitotic metaphase without chromosome measurements — with the use of just one molecular probe that is (TTTAGGG)n telomeric sequence (Fig. 4). Thus, the structure of chromosome arms that conjoin in the meiotic ring can be now much more easily studied than ever before using cytologically preferable mitotic material. Furthermore, the chromosomes do not need to be reprobed to analyze the chromosomal arrangement of another DNA sequence on the identified chromosomal arms.

### Repetitive DNAs of interstitial chromosome regions as parts of non-recombining chromatin

The structural heterozygosity of the intercalary chromosomal sections in oyster plants may suppress crossing-over within relevant genomic subregions, as in many other organisms (reviewed in: Huang and Rieseberg 2020; Gutiérrez-Valencia et al. 2021). An assemblage of structural heterozygosities for inversions/indels creates unpaired DNA loops preventing recombination (Torgasheva and Borodin 2010; Huang and Rieseberg 2020). Actually, even an interruption in the continuity of the synaptonemal complex (SC) at the site of a breakpoint can halt recombination over a long distance from the breakpoint (Gong et al. 2005). Indeed, Stack and Soulliere (1984) demonstrated that telomere-led synapsis in the oyster plant is confined to distal segments of a size equal to 20–30% of the chromosomal length. Notably, the distance from the chromosome termini to the nearest intercalary repetitive loci, which we termed here “distal section” (Fig. 4) fits 20–30% of the chromosomal length (Table S2 and explanations therein). Consequently, our data strongly suggests the importance of the repetitive DNA sequences for the evolution and properties of non-recombining euchromatin in the oyster plant.

Since euchromatic regions with high meiotic recombination rates tend to be enriched with CA/GT repetitive tracts (Cuadrado et al. 2008 and references therein; Guo et al., 2009 and references therein), one may predict that that in a PTH organism only subtelomeric (recombining) regions will show strong labeling, as opposed to remaining genomic regions. Thus, the weak labeling or its absence were expected by us to be correlated at least with structurally heterozygous intercalary regions and with pericentromeric heterochromatin. This however was not the case, since all the euchromatin showed rather strong but uniform (CA)_10_ FISH labeling (Fig. 3d). Thus, the lack of CA/GT dinucleotide in oyster plant is rather related to the pericentromere/centromere functions/peculiarities rather than to a non-recombining PTH genomic regions per se. For example, the exclusion of the CA/GT clusters from pericentromeric heterochromatin may be due to a purifying selection securing the genome from overdosing on general centromere instability conferred by microsatellites (Guo et al. 2009).

### Inversions and whole arm translocations as mechanisms responsible for intra- and interchromosomal DNA movement of repetitive DNA

The shared occurrence of 45S rDNA and telomeric sequences at a site where neither of them usually resides is decidedly viewed as an indicator of chromosomal rearrangements (Raskina et al. 2008; Grabowska-Joachimiak et al. 2015). The concerted spread of the telomeric motif, 45S rDNA, and TSrepI repeats throughout all the twelve pericentromeric regions suggests the occurrence of pericentromeric breakpoints that generated whole arm translocations via ectopic recombination between clustered pericentromeres of the Rabl configuration. Some pericentromeric breakpoint sites, like eu-heterochromatin transitions which are a junction between the early and late replicating regions in the oyster plant (Natarajan and Natarajan 1972), might have been however more frequently used than others (Chandley 1986). Whether the TTTAGGG-enriched external parts of the pericentromeric heterochromatin on chromosomes 5, 6, and 8 (Figs. 2 and 4) are the footprints of the “hottest” breakpoints remains to be resolved. Such genomic regions may arise via segmental duplications that increase sequence identity between breakpoint sites, rendering further rearrangements involving these sites even more likely (Eichler 1999; Bulazel et al. 2007).

Since the three DNA repeats are present also in distal chromosome regions, subtelomere-pericentromere sequence movement via arm inversions may have occurred. Inversions are pervasive features of the best-studied non-PTH supergenes, being able to span entire chromosomes with dynamics similar to that known for sex chromosomes (Schwander et al. 2014). Together with reciprocal translocations, they constitute the core of the PTH system in *Paeonia californica* and *Paeonia brownie* (Snow 1969). Referring to the oyster plant, Darlington (1937, p. 279) wrote: “Within its differential segments must be many inversions, yet these never pair.” A start-up installation of recombinogenic tandem repeats like rDNA or telomeric motif (Huang et al. 2012; Grabowska-Joachimiak et al. 2015) into the pericentromeric area by whole-arm inversions may have increased pericentromere fragility, provoking whole arm translocations and further inversions, with the resultant genome-wide concerted sequence migration.

**Fig. 5:**
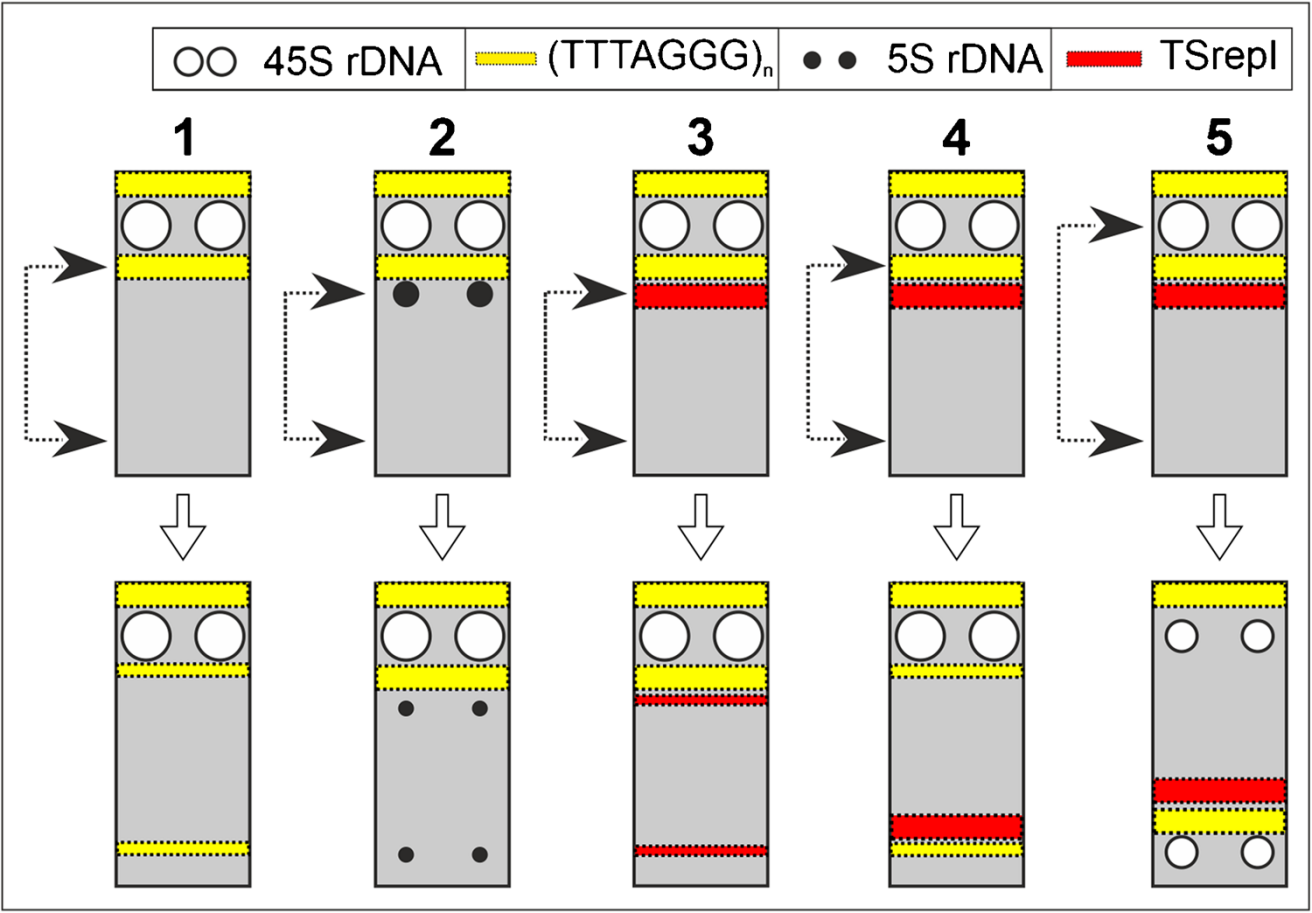
Complex structure of a subtelomeric region and variability in repetitive sequence movement via inversion. If a breakpoint (upper arrowhead) occurs within the innermost subtelomeric repetitive locus (1, 2, 3), an effect is jumping of its part away from the subtelomere into a more internal region close to the second breakpoint (lower arrowhead). The joint movement of different types of repetitive DNA occurs if the breakpoint occurs within a subtelomeric repetitive sequence cluster that is positioned distally to the innermost locus (4, 5). For simplicity, centromere and the second subtelomeric region were not depicted here

On the other hand, the disjoint spread of DNA repeats along the chromsomal arms and the lack of detectable 5S rDNA sites in the pericentromeric area may be due to degeneration/elimination processes and/or due to a complex structure of distal chromosome regions where inversion breakpoints may yield different results depending on localization (Fig. 5).

Duplications of (TTTAGGG)_n_ and 5S rDNA loci (Fig. 4) may reflect a general trend for the sequence movement via inversions, i.e., paracentric inversions with one breakpoint within the already existing locus. The two interstitial minor 5S rDNA loci on chromosome arms 3b and 4b (Figs. 3f and 4) may have resulted via a large ancient segmental inversion, involving ca. 40% of the arm length, with one of the breakpoints within the subtelomeric major 5S rDNA locus. The occurrence of inversions in b arms and in chromosomes 9–10 was inferred by Straub (1941) and by Flagg (1958), respectively. Interestingly, the uncovered homozygous or partially homozygous condition of some interstitial regions (Fig. 4) may suggest that inversions started to form linkages already in a bivalent-forming ancestor as a preparatory step towards restricted recombination and PTH. The restriction of recombination to chromosomal ends in *Oenothera*, including bivalent-forming ancestral species, suggests that it was an early evolutionary event that might have been caused by ancient chromosomal changes different from reciprocal translocations, affecting ancestral bivalent-forming species and facilitating further evolution of reciprocal translocations and, consequently, meiotic rings (Rauwolf et al. 2011).

### Rabl configuration for PTH

The strongly reshuffled PTH genomes express an extraordinary high degree of nuclear order and compartmentation (Golczyk et al. 2014, 2020). Both in the oyster plant and in *Oenothera* species, pericentromeric regions remain clustered and generate strong Rabl-polarization in cells of the root tip meristem and during pachytene (Golczyk 2011b, c; Golczyk et al. 2005, 2008, 2014, 2020). Moreover, in the oyster plant, strikingly, regular non-random Rabl-polarized spatial chromatin dispositions were observed, including rings or chains of side-by-side juxtaposed pericentromeres in somatic interphase and during pachytene (Golczyk et al. 2020). Clustering of pericentromeric regions and the strong telomere-centromere polarization may have been an excellent framework for the whole arm translocations, subtelomere-pericentromere sequence migration and the resultant homogenization of all the pericentromeric domains by subtelomeric sequences (see Introduction). Indeed, we showed here that the pericentromeric regions of the oyster plant are invaded in concert by three subtelomeric sequences (see “Inversions and whole arm translocations as mechanisms responsible for intra- and interchromosomal DNA movement of repetitive DNA”).

However, a non-random Rabl-based spatial genome organization has been also suggested by earlier authors to support the nucleus with environment where sites equally distant from the centromere pole such as distal regions of similarly-sized chromosomal arms, contact and share their repetitive sequences (Schweizer and Loidl 1987). Complying with such a Rabl-based repetitive DNA spread, we could demonstrate that chromosome arms unequal in length differ in the subtelomeric sequence composition. In particular, the subterminal TSrepI loci reside exclusively on the longer arms. Thus, this data adds another interesting potential cytomolecular link to the concept that the evolution of PTH in the oyster plant may have been affected by Rabl arrangement. This inspiring idea deserves comprehensive verification in the future that should include mapping more repetitive sequences and use them to test the somatic alignment of equally sized chromosomal arms in the nucleus.

On the other hand, the remarkable potential of the oyster plant for Rabl-based nuclear constraints is likely to be also of a functional importance, presumably because it may help in regular pairing and disjunction in face of multiple homologies and other structural complications on chromosomes that resulted from extensive rearrangements. Indeed, the untypical aggregation of pericentromeric heterochromatin (from leptotene until late pachytene) coupled with the strong centromere-telomere polarization in the oyster plant (Golczyk 2011b, c; Stack and Soulliere 1984; Golczyk et al. 2020) resembles the meiotic behavior in allopolyploids (Naranjo and Corredor 2004). This similarity to hybrid species does suggest the potential danger of extensive homology search errors that could prevent proper meiotic pairing and a regular segregation of the Renner complexes.

## Supplementary Information

Below is the link to the electronic supplementary material.

Supplementary file1 (DOCX 43 KB)

Supplementary file2 (DOCX 20 KB)

ESM 3 Data Set S 1 TSrepI.01 sequence in the FASTA format (FAS 446 bytes)

ESM 4 Fig. S 1 Dot-plot analysis of the TSrepI.01 sequence. Degenerated semi-tandemly organized units within the entire sequence are visible. Similarities are represented by dots or lines. (PNG 75 kb)

High resolution image (TIF 6166 kb)

## Abbreviations

PTH: Permanent translocation heterozygosity
FISH: Fluorescent in situ hybridisation
rDNA: Ribosomal DNA
