# Supplementary material for: Migration of repetitive DNAs during evolution of the permanent translocation heterozygosity in the oyster plant (Tradescantia section Rhoeo)
Source: Chromosoma. 2022 Jul 27;131(3):163–73. doi: 10.1007/s00412-022-00776-1 (PMC9470650; doi:10.1007/s00412-022-00776-1)
Supplement: Supplementary file 1 — Supplementary file1 (DOCX 43 KB) [file 412_2022_776_MOESM1_ESM.docx]

**Table S1.**

Intercalary and distal sections distinguished for eight “pairs” of chromosomes (1-2, 3-4, 4-5, 6-7, 7-8 9-10, 10-11, 12-1) and their arms conjoining in the ring (e.g., 1a-2a, etc.)*.* Section size expressed as the percentage of: chromosome length (ChL), arm length (AL), karyotype length (KL). (hom, het, hom/het), structurally homozygous (hom), heterozygous (het), or partially homozygous/heterozygous intercalary sections (hom/het). Standard deviations within brackets are placed under the mean values. *****

|  |  |  | |  |  |  | |  |  | |  |  | |  |  | |  |  | |  |  | | |  |  | |  |  |
| --- | --- | --- | --- | --- | --- | --- | --- | --- | --- | --- | --- | --- | --- | --- | --- | --- | --- | --- | --- | --- | --- | --- | --- | --- | --- | --- | --- | --- |
|  |  | ___1-2___ | | |  | ___3-4___ | |  | ___4-5___ | |  | ___6-7___ | |  | ___7-8___ | |  | ___9-10___ | |  | ___10-11___ | | |  | ___12-1___ | |  | total |
|  |  | 1a | | 2a |  | 3b | 4b |  | 4C | 5C |  | 6D | 7D |  | 7d | 8d |  | 9e | 10e |  | 10F | | 11F |  | 12A | 1A |  |  |
|  |  |  | |  |  |  |  |  |  |  |  |  |  |  |  |  |  |  |  |  |  | |  |  |  |  |  |  |
| intercalary  sections  ( I ) | ChL | 21.05  (0.82) | | 26.77  (1.86) |  | 25.86  (1.66) | 20.92  (0.90) |  | 23.80  (1.10) | 25.92  (2.28) |  | 24.50  (1.38) | 24.93  (1.31) |  | 18.50  (1.29) | 17.62  (1.92) |  | 15.15  (1.35) | 12.84  (1.10) |  | 17.95  (1.02) | | 20.76  (1.54) |  | 26.03  (1.27) | 21.66  (1.76) |  |  |
|  |  |  | |  |  |  |  |  |  |  |  |  |  |  |  |  |  |  |  |  |  | |  |  |  |  |  |  |
|  | AL | 46.52  (1.82) | | 39.03  (2.71) |  | 36.17  (2.32) | 39.91  (1.71) |  | 49.82  (2.30) | 36.99  (3.25) |  | 38.65  (2.18) | 61.49  (3.23) |  | 31.21  (2.18) | 27.85  (3.03) |  | 22.52  (2.01) | 25.47  (2.19) |  | 36.31  (2.06) | | 37.41  (2.78) |  | 44.75  (2.18) | 39.66  (3.23) |  |  |
|  |  |  | | |  |  | |  |  | |  |  | |  |  | |  |  | |  |  | | |  |  | |  |  |
|  | KL | 2.08 (0.12) | 2.08 (0.14) | |  | 2.10 (0.13) | 2.10 (0.09) |  | 2.39 (0.11) | 2.39 (0.20) |  | 1.86 (0.10) | 1.86 (0.09) |  | 1.38 (0.11) | 1.38 (0.15) |  | 1.13 (0.12) | 1.13 (0.09) |  | 1.58 (0.08) | 1.58 (0.11) | |  | 2.14 (0.10) | 2.14 (0.18) |  | 29.32  (2.44) |
|  |  | hom | | |  | hom / het | |  | het | |  | het | |  | het | |  | het | |  | hom / het | | |  | het | |  |  |
|  |  |  | | |  |  | |  |  | |  |  | |  |  | |  |  | |  |  | | |  |  | |  |  |
|  |  |  | | |  |  | |  |  | |  |  | |  |  | |  |  | |  |  | | |  |  | |  |  |
|  |  |  | | |  |  | |  |  | |  |  | |  |  | |  |  | |  |  | | |  |  | |  |  |
| distal  sections | ChL | 11.54  (0.46) | | 14.67  (1.02) |  | 16.50  (1.06) | 13.35  (0.57) |  | 7.97  (0.37) | 8.68  (0.76) |  | 5.27  (0.30) | 5.36  (0.28) |  | 24.40  (1.70) | 23.24  (2.53) |  | 24.53  (2.19) | 20.79  (1.79) |  | 9.77  (0.56) | | 11.30  (0.84) |  | 14.96  (0.73) | 12.45  (1.01) |  |  |
|  |  |  | |  |  |  |  |  |  |  |  |  |  |  |  |  |  |  |  |  |  | |  |  |  |  |  |  |
|  | AL | 25.51  (1,01) | | 21.43  (1.49) |  | 22.95  (1.47) | 25.92  (1.11) |  | 16.37  (0.76) | 12.73  (1.12) |  | 8.24  (0.46) | 13.50  (0.71) |  | 40.84  (2.85) | 36.86  (4.01) |  | 36.65  (3.27) | 41.80  (3.59) |  | 19.74  (1.12) | | 20.72  (1.54) |  | 25.09  (1.22) | 23.45  (1.91) |  |  |
|  |  |  | | |  |  | |  |  | |  |  | |  |  | |  |  | |  |  | | |  |  | |  |  |
|  | KL | 1.14 (0.05) | 1.14 (0.08) | |  | 1.34 (0.09) | 1.34 (0.06) |  | 0.80 (0.04) | 0.80 (0.07) |  | 0.40 (0.03) | 0.40 (0.02) |  | 1.82 (0.13) | 1.82 (0.20) |  | 1.83 (0.16) | 1.83 (0.14) |  | 0.86 (0.05) | 0.86 (0.06) | |  | 1.23 (0.07) | 1.23 (0.10) |  | 18.84 (1.64) |
|  |  |  | | |  |  | |  |  | |  |  | |  |  | |  |  | |  |  | | |  |  | |  |  |

***** Intercalary sections occupy 29.32 % of the karyotype. Among them are: five pairs of structurally heterozygous sections (17.8%), one pair of structurally homozygous sections (4.16%) and two pairs whose members are partially homozygous/heterozygous (7.36%). Individual sections are quite large – from ca. 22% to 61%, if their size is expressed as the percentage of the chromosomal armes bearing them (AL). Distal sections constitute 18.84 % of the karyotype. For graphical illustration, see Figure 4 in the main text.
