# Supplementary material for: Migration of repetitive DNAs during evolution of the permanent translocation heterozygosity in the oyster plant (Tradescantia section Rhoeo)
Source: Chromosoma. 2022 Jul 27;131(3):163–73. doi: 10.1007/s00412-022-00776-1 (PMC9470650; doi:10.1007/s00412-022-00776-1)
Supplement: Supplementary file 2 — Supplementary file2 (DOCX 20 KB) [file 412_2022_776_MOESM2_ESM.docx]

**Table S2.**

The distal chromosomal sections in the oyster plant correlate in size with the model of restricted synapsis of Stack and Soulliere (1984).

Size of distal chromosomal sections in *T. spathacea* expressed as the percentage of chromosomal length or of karyotype length. Values are means from Supplemental Table 1.

|  |  |  |  |  |  |  | |  |  |  |
| --- | --- | --- | --- | --- | --- | --- | --- | --- | --- | --- |
|  |  |  | distal sections | | | | | | | |
|  |  |  |  | | |  | |  | | |
|  |  |  |  | | |  | |  | | |
|  |  |  | % of chromosome length | | |  | | % of karyotype length | | |
|  |  |  |  |  |  |  | |  |  |  |
|  |  |  |  |  |  |  | |  |  |  |
| 1 * | 1A |  | 12.45 |  | 23.99 |  | | 1.23 |  | 2.37 |
|  | 1a |  | 11.54 |  |  |  | | 1.14 |  |  |
|  |  |  |  |  |  |  | |  |  |  |
| 2 | 2a |  | 14.67 |  | 14.67 |  | | 1.14 |  | 1.14 |
|  |  |  |  |  |  |  | |  |  |  |
| 3 | 3b |  | 16.50 |  | 16.50 |  | | 1.34 |  | 1.34 |
|  |  |  |  |  |  |  | |  |  |  |
| 4 * | 4b |  | 13.35 |  | 21.32 |  | | 1.34 |  | 2.14 |
|  | 4C |  | 7.97 |  |  |  | | 0.80 |  |  |
|  |  |  |  |  |  |  | |  |  |  |
| 5 | 5C |  | 8.68 |  | 8.68 |  | | 0.80 |  | 0.80 |
|  |  |  |  |  |  |  | |  |  |  |
| 6 | 6D |  | 5.27 |  | 5.27 |  | | 0.40 |  | 0.40 |
|  |  |  |  |  |  |  | |  |  |  |
| 7 * | 7D |  | 5.36 |  | 29.76 |  | | 0.40 |  | 2.22 |
|  | 7d |  | 24.40 |  |  |  | | 1.82 |  |  |
|  |  |  |  |  |  |  | |  |  |  |
| 8 | 8d |  | 23.24 |  | 23.24 |  | | 1.82 |  | 1.82 |
|  |  |  |  |  |  |  | |  |  |  |
| 9 | 9e |  | 24.53 |  | 24.53 |  | | 1.83 |  | 1.83 |
|  |  |  |  |  |  |  | |  |  |  |
| 10 * | 10e |  | 20.79 |  | 30.56 |  | | 1.83 |  | 2.69 |
|  | 10F |  | 9.77 |  |  |  | | 0.86 |  |  |
|  |  |  |  |  |  |  | |  |  |  |
| 11 | 11F |  | 11.30 |  | 11.30 |  | | 0.86 |  | 0.86 |
|  |  |  |  |  |  |  | |  |  |  |
| 12 | 12A |  | 14.96 |  | 14.96 |  | | 1.23 |  | 1.23 |
|  |  |  |  |  |  |  | |  |  |  |
| total % of karyotype length | | | | | | | 18.84 | | | |
|  |  |  |  |  |  |  | |  |  |  |

Chromosomes: 1, 4, 7 and 10 (asterisks) serve as a reference since each of them has a distal section on either arm. The summed-up length of the two distal sections belonging to any of these chromosomes is within 21-31% of the chromosomal size. Consequently, distal sections well correlate in length with the synapsing distal regions, at least for these four chromosomes. If percentage of the karyotype length is used, the average value is 1.18 % of the karyotype per one distal section. Thus, the simulated total 28.32% (24 x 1.18%) still fits well to the 20-30% of the diploid genome engaged in effective pairing, as predicted by Stack and Soulliere. That the calculated value is reliable can be seen if all the distal sections are summed up (we get 18.84 %) and then divided by 16 (in total 16 distal sections are distinguished), which gives again 1.18 % of the karyotype per one distal section.
