# Supplementary figures and images for: Migration of repetitive DNAs during evolution of the permanent translocation heterozygosity in the oyster plant (Tradescantia section Rhoeo)
Source: Chromosoma. 2022 Jul 27;131(3):163–73. doi: 10.1007/s00412-022-00776-1 (PMC9470650; doi:10.1007/s00412-022-00776-1)

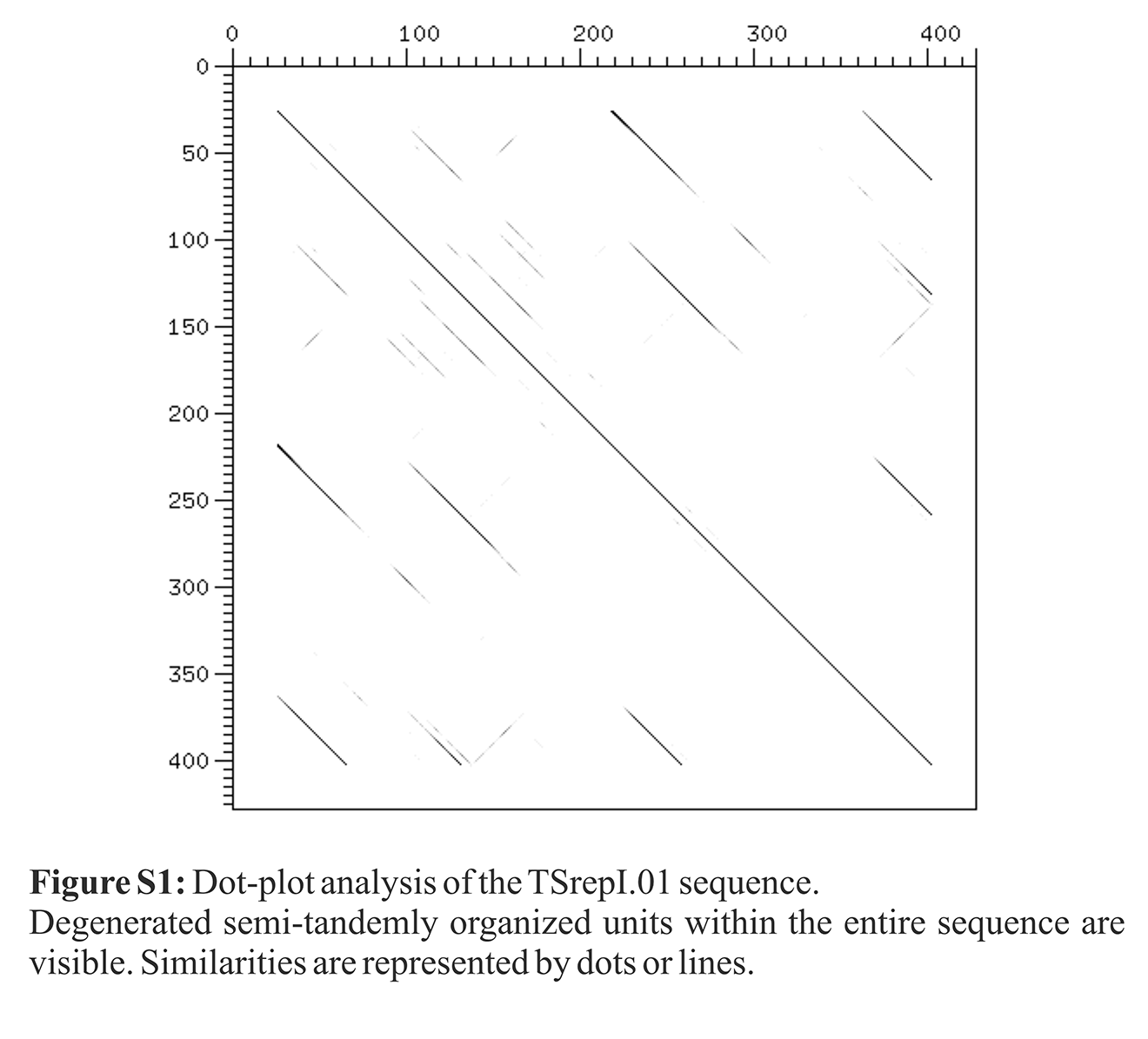

Supplement: Supplementary file 4 — Fig. S 1 Dot-plot analysis of the TSrepI.01 sequence. Degenerated semi-tandemly organized units within the entire sequence are visible. Similarities are represented by dots or lines. (PNG 75 kb) [file 412_2022_776_Fig6_ESM.png]

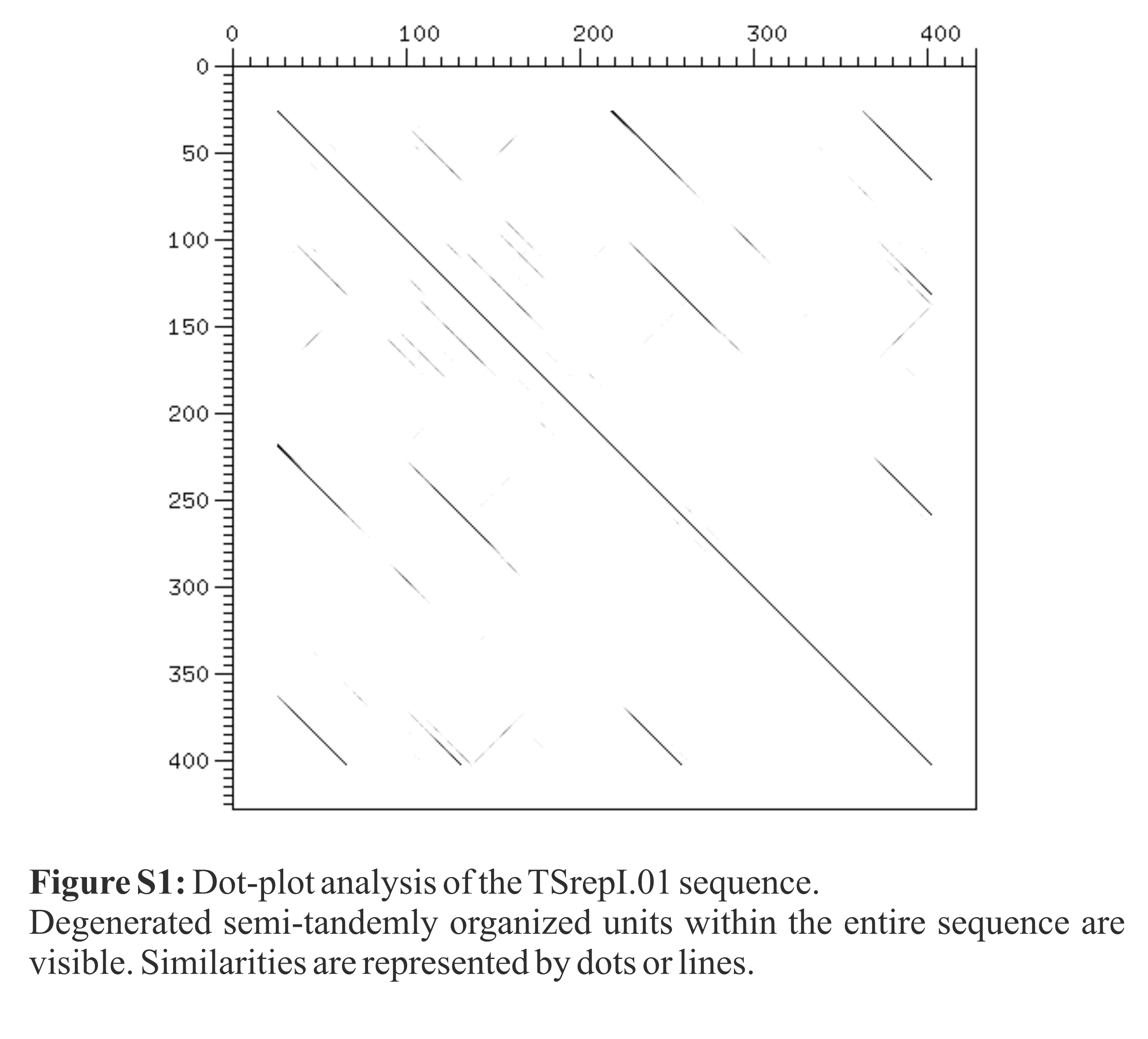

Supplement: Supplementary file 5 — High resolution image (TIF 6166 kb) [file 412_2022_776_MOESM4_ESM.tif]
